# Phylogeography of the Japanese greater horseshoe bat *Rhinolophus nippon* (Mammalia: Chiroptera) in Northeast Asia: New insight into the monophyly of the Japanese populations

**DOI:** 10.1002/ece3.8414

**Published:** 2021-12-03

**Authors:** Yugo Ikeda, Masaharu Motokawa

**Affiliations:** ^1^ Graduate School of Science Kyoto University Sakyo Kyoto Japan; ^2^ Kyoto University Museum Kyoto University Sakyo Kyoto Japan

**Keywords:** greater horseshoe bat, island biology, land bridge, phylogeny, reverse colonization

## Abstract

The Japanese greater horseshoe bat (*Rhinolophus nippon*) is distributed widely in East Asia. Within the species, *R*. *nippon* in Northeast Asia is regarded as the lineage that diverged most recently. However, the monophyly of the Japanese populations is unclear due to insufficient data about phylogenetic relationship of the western Japanese populations. To test the monophyly of the Japanese populations of *R*. *nippon*, we sampled *R*. *nippon* from western Japan and performed a phylogeographic analysis based on mitochondrial DNA cytochrome *b* and the D‐loop. The Northeast Asian lineage consisted of three main clades in eastern Japan (clade I), western Japan (clade II), and the continent as well as the Kumamoto population in westernmost Japan (clade III). The results of this study do not support the monophyly of the Japanese population. The findings suggest the “reverse colonization” of *R*. *nippon* from the Japanese Archipelago to the Eurasian continent, and provide important insight into the role of the island system in creation and supply of diversity to the continent.

## Graphical abstract

We performed a phylogeographic analysis of the Japanese greater horseshoe bat (*Rhinolophus nippon*) in Northeast Asia based on mitochondrial DNA cytochrome *b* and the D‐loop. The results revealed that the Japanese populations are not monophyletic, and the continental populations are imbedded within the Japanese population. The findings support the "reverse colonization" of *R. nippon* from the Japanese Archipelago to the Eurasian continent.

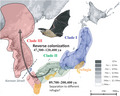

## 1 INTRODUCTION

The bat order Chiroptera (Mammalia) is the second most speciose order of mammals, consisting of at least 21 families, 230 genera, and 1401 species (Wilson & Mittermeier, 2019). It is distributed worldwide, including on various islands, but not in the polar regions. Evolutionary biological studies of organisms on islands—island biological studies—have focused on birds that evolved dramatically as the result of isolation and adaptive radiation to the island environment as represented by Darwin's finches (Burns et al., 2002; Harvey et al., 2021; Lack, 1945; Lamichhaney et al., 2016; Sato et al., 1999; Weir et al., 2009). Although bats are regular members of island ecosystems and adapted to island environments, they have been rarely focused in island biological research.

The greater horseshoe bat *Rhinolophus ferrumequinum* complex (Chiroptera: Rhinolophidae), which occurs throughout the Palearctic region, including many islands, had been considered a single species until recently (Csorba et al., 2003; Huston et al., 2019; Jo et al., 2018; Sano, 2015; Smith, 2008; Yoshiyuki, 1989). Based on molecular studies (Flanders et al., 2009, 2011; Koh et al., 2014; Rossiter et al., 2007), the European greater horseshoe bat *R*. *ferrumequinum* (Schreber, 1774) in the western Palearctic and the Japanese greater horseshoe bat *R*. *nippon* Temminck, 1835 in the eastern Palearctic became recognized as separate species (Burgin, 2019), which were subsequently diagnosed from each other and redescribed based on skull morphological characters (Ikeda, Jiang, et al., 2020). However, unresolved taxonomic problems remain for *R*. *nippon* populations in Northeast Asia, which includes northeastern China (Jilin and Liaoning provinces), the Korean Peninsula, the Japanese Archipelago, and peripheral islands.

The Japanese Archipelago is a biodiversity hotspot with many endemic species of various mammals including bats (Motokawa, 2015). In contrast, the distributions of several species of bats, including *R*. *nippon*, extend to the Eurasian continent. Many Japanese terrestrial animals are considered to have origin in the Eurasian continent and to have migrated through the Korean Peninsula (*e*.*g*., Tamate, 2015). When discussing the origins of Japanese terrestrial animals, phylogeographic patterns among populations in Japan and Northeast Asia are necessary to be clarified.

Flanders et al. (2011) reported that *R*. *nippon* populations in Northeast Asia (from Jilin Province and eastern Japan) diverged deeply from the parapatric populations in East China (Henan Province) and form a monophyletic group based on 1098 bp of the mitochondrial *ND2* gene and 13 microsatellite loci. These data suggest that the Northeast Asian lineage diverged 400,000–600,000 years ago. Then, Liu et al. (2016) revealed that the Japanese populations form a monophyletic clade that diverged from the continental populations (from Jilin and Liaoning provinces and South Korea) more recently, based on mtDNA cytochrome *b* data. These two studies included Japanese samples only from eastern Honshu, and did not examine the western Japanese samples (western Honshu, Kyushu, and Shikoku islands). Honshu is the largest island in East Asia and several species showed unexpected divergence within the island (Motokawa, 2017). In fact, terrestrial animals in the Japanese Archipelago tend to be diverged two or more lineages in east and west. Therefore, we conducted molecular phylogeographic analyses of Northeast Asian *R*. *nippon*, including the western Japanese populations, to verify the monophyly of the Japanese populations.

## 2 MATERIALS AND METHODS

### 2.1 Specimens and sampling

**FIGURE 1:**
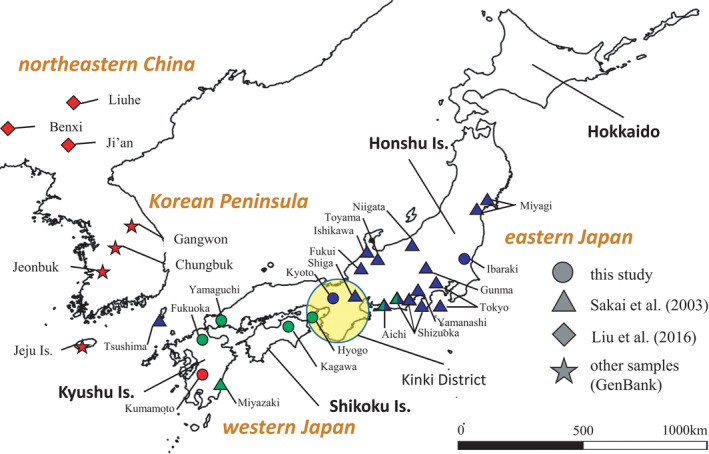
Map of the Northeast Asian *Rhinolophus nippon* samples examined in this study. Colors of an object represent clades: blue, clade I; green, clade II; red, clade III. Circles represent samples collected in this study; Triangles, diamonds, and stars represent samples reported in Liu et al. (2016), Sakai et al. (2003) and deposited in GenBank, respectively

Thirty‐two *R*. *nippon* specimens and one *R*. *cornutus* specimen were collected from seven localities in Japan: Ibaraki, Kyoto, Hyogo, and Yamaguchi on Honshu Island, Kagawa on Shikoku Island, and Fukuoka and Kumamoto on Kyushu Island (Figure 1). All specimens were deposited in the Zoological Collection of Kyoto University (KUZ M17251–KUZ M17283). To verify the consistency with Liu et al. (2016), we targeted the cytochrome *b* and the D‐loop mitochondrial DNA (mtDNA) regions; the latter has a high evolutionary rate and is suitable for intraspecific comparison. Thirty‐five sequences from eastern Japan (cytochrome *b* alleles by Sakai et al. [2003]), South Korea, and China deposited in GenBank and 20 sequences from Jilin (Ji'an and Liuhe) and Liaoning (Benxi) provinces reported by Liu et al. (2016) were included in the phylogenetic analyses (Table 1, Figure 1). In haplotype and nucleotide diversities and neutrality tests, the sequence data of Sakai et al. (2003) were treated as the actual number of individuals.

**TABLE 1:** Characteristics of the samples examined in this study

No.	ID	Accession		Haplotype	Clade	*n*	Country	Region	Locality	Latitude	Longitude	Altitude	Note
		cyt *b*	D–loop										
1	KUZM17251	LC605914	LC605947	H1‐1	I	1	Japan	eastern Japan	Kyoto	35.24	135.49	276	–
2	KUZM17252	LC605915	LC605948	H1‐1	I	1	Japan	eastern Japan	Kyoto	35.24	135.49	276	–
3	KUZM17253	LC605916	LC605949	H1‐1	I	1	Japan	eastern Japan	Kyoto	35.24	135.49	276	–
4	KUZM17254	LC605917	LC605950	H2‐1	I	1	Japan	eastern Japan	Kyoto	35.24	135.49	276	–
5	KUZM17255	LC605918	LC605951	H3‐1	III	1	Japan	western Japan	Kumamoto	32.22	130.82	150	–
6	KUZM17256	LC605919	LC605952	H4‐1	III	1	Japan	western Japan	Kumamoto	32.22	130.82	150	–
7	KUZM17257	LC605920	LC605953	H4‐1	III	1	Japan	western Japan	Kumamoto	32.22	130.82	150	–
8	KUZM17258	LC605921	LC605954	H4‐1	III	1	Japan	western Japan	Kumamoto	32.22	130.82	150	–
9	KUZM17259	LC605922	LC605955	H3‐2	III	1	Japan	western Japan	Kumamoto	32.41	130.87	289	–
10	KUZM17260	LC605923	LC605956	H3‐3	III	1	Japan	western Japan	Kumamoto	32.41	130.87	289	–
11	KUZM17261	LC605924	LC605957	H5‐1	II	1	Japan	western Japan	Fukuoka	33.79	130.94	321	–
12	KUZM17262	LC605925	LC605958	H5‐1	II	1	Japan	western Japan	Fukuoka	33.79	130.94	321	–
13	KUZM17263	LC605926	LC605959	H6‐1	II	1	Japan	western Japan	Fukuoka	33.79	130.94	321	–
14	KUZM17264	LC605927	LC605960	H6‐2	II	1	Japan	western Japan	Fukuoka	33.79	130.94	321	–
15	KUZM17265	LC605928	LC605961	H6‐3	II	1	Japan	western Japan	Yamaguchi	34.25	131.25	117	–
16	KUZM17266	LC605929	LC605962	H6‐4	II	1	Japan	western Japan	Yamaguchi	34.25	131.25	117	–
17	KUZM17267	LC605930	LC605963	H6‐3	II	1	Japan	western Japan	Yamaguchi	34.25	131.25	104	–
18	KUZM17268	LC605931	LC605964	H6‐3	II	1	Japan	western Japan	Yamaguchi	34.25	131.25	117	–
19	KUZM17269	LC605932	LC605965	H6‐3	II	1	Japan	western Japan	Yamaguchi	34.25	131.25	117	–
20	KUZM17270	LC605933	LC605966	H1‐2	I	1	Japan	eastern Japan	Ibaraki	36.42	140.37	90	–
21	KUZM17271	LC605934	LC605967	H1‐3	I	1	Japan	eastern Japan	Ibaraki	36.19	140.15	113	–
22	KUZM17272	LC605935	LC605968	H7‐1	II	1	Japan	western Japan	Hyogo	34.25	134.81	193	–
23	KUZM17273	LC605936	LC605969	H8‐1	II	1	Japan	western Japan	Hyogo	34.25	134.81	193	–
24	KUZM17274	LC605937	LC605970	H7‐1	II	1	Japan	western Japan	Hyogo	34.25	134.81	193	–
25	KUZM17275	LC605938	LC605971	H7‐1	II	1	Japan	western Japan	Hyogo	34.25	134.81	193	–
26	KUZM17276	LC605939	LC605972	H9‐1	II	1	Japan	western Japan	Kagawa	34.16	133.89	211	–
27	KUZM17277	LC605940	LC605973	H10‐1	II	1	Japan	western Japan	Kagawa	34.16	133.89	211	–
28	KUZM17278	LC605941	LC605974	H11‐1	II	1	Japan	western Japan	Kagawa	34.16	133.89	211	–
29	KUZM17279	LC605942	LC605975	H1‐4	I	1	Japan	eastern Japan	Kyoto	35.20	135.65	–	–
30	KUZM17280	LC605943	LC605976	H1‐5	I	1	Japan	eastern Japan	Kyoto	35.20	135.65	–	–
31	KUZM17281	LC605944	LC605977	H1‐6	I	1	Japan	eastern Japan	Kyoto	35.20	135.65	–	–
32	KUZM17282	LC605945	LC605978	H1‐1	I	1	Japan	eastern Japan	Kyoto	35.20	135.65	–	–
33	–	JA1	JA1	H3‐4	III	1	NE China	Jilin	Ji'an	41.05	125.83	–	Appendix data of Liu et al. (2016)
34	–	JA2	JA2	H3‐4	III	1	NE China	Jilin	Ji'an	41.05	125.83	–	Appendix data of Liu et al. (2016)
35	–	JA3	JA3	H3‐4	III	1	NE China	Jilin	Ji'an	41.05	125.83	–	Appendix data of Liu et al. (2016)
36	–	JA4	JA4	H3‐4	III	1	NE China	Jilin	Ji'an	41.05	125.83	–	Appendix data of Liu et al. (2016)
37	–	JA6	JA6	H3‐4	III	1	NE China	Jilin	Ji'an	41.05	125.83	–	Appendix data of Liu et al. (2016)
38	–	JA7	JA7	H3‐4	III	1	NE China	Jilin	Ji'an	41.05	125.83	–	Appendix data of Liu et al. (2016)
39	–	JA8	JA8	H3‐4	III	1	NE China	Jilin	Ji'an	41.05	125.83	–	Appendix data of Liu et al. (2016)
40	–	JA9	JA9	H3‐4	III	1	NE China	Jilin	Ji'an	41.05	125.83	–	Appendix data of Liu et al. (2016)
41	–	JA11	JA11	H3‐4	III	1	NE China	Jilin	Ji'an	41.05	125.83	–	Appendix data of Liu et al. (2016)
42	–	JA12	JA12	H3‐5	III	1	NE China	Jilin	Ji'an	41.05	125.83	–	Appendix data of Liu et al. (2016)
43	–	JA13	JA13	H3‐4	III	1	NE China	Jilin	Ji'an	41.05	125.83	–	Appendix data of Liu et al. (2016)
44	–	JA14	JA14	H3‐6	III	1	NE China	Jilin	Ji'an	41.05	125.83	–	Appendix data of Liu et al. (2016)
45	–	JA15	JA15	H3‐6	III	1	NE China	Jilin	Ji'an	41.05	125.83	–	Appendix data of Liu et al. (2016)
46	–	JA16	JA16	H3‐6	III	1	NE China	Jilin	Ji'an	41.05	125.83	–	Appendix data of Liu et al. (2016)
47	–	LH1	LH1	H12‐1	III	1	NE China	Jilin	Liuhe	42.38	126.00	–	Appendix data of Liu et al. (2016)
48	–	LH3	LH3	H3‐4	III	1	NE China	Jilin	Liuhe	42.38	126.00	–	Appendix data of Liu et al. (2016)
49	–	LH4	LH4	H12‐1	III	1	NE China	Jilin	Liuhe	42.38	126.00	–	Appendix data of Liu et al. (2016)
50	–	JN392460	JN392460	H3‐4	III	1	South Korea	Korean Peninsula	Gangwon	–	–	–	complete genome
51	–	JX084273	JX084273	H3‐4	III	1	South Korea	Jeju	Jeju	–	–	–	complete genome
52	–	NC020326	NC020326	H3‐4	III	1	South Korea	Jeju	Jeju	–	–	–	complete genome
53	–	NC016191	NC016191	H3‐4	III	1	South Korea	Korean Peninsula	Gangwon	–	–	–	complete genome
54	–	KT779432	KT779432	H3‐5	III	1	NE China	Jilin	–	–	–	–	complete genome
55	–	KT783534	KT783534	H36	SW China	1	SW China	Yunnan	–	–	–	–	complete genome
56	–	BX11	–	H3	III	1	NE China	Liaoning	Benxi	41.38	124.95	–	Appendix data of Liu et al. (2016)
57	–	BX12	–	H3	III	1	NE China	Liaoning	Benxi	41.38	124.95	–	Appendix data of Liu et al. (2016)
58	–	BX13	–	H3	III	1	NE China	Liaoning	Benxi	41.38	124.95	–	Appendix data of Liu et al. (2016)
59	–	KP063140	–	H13	III	1	South Korea	Jeju	Jeju	–	–	–	–
60	–	KP063141	–	H14	III	1	South Korea	Jeju	Jeju	–	–	–	–
61	–	KP063142	–	H15	III	1	South Korea	Jeju	Jeju	–	–	–	–
62	–	KP063143	–	H16	III	1	South Korea	Korean Peninsula	Jeonbuk	–	–	–	–
63	–	KP063144	–	H17	III	1	South Korea	Korean Peninsula	Chungbuk	–	–	–	–
64	–	KP063145	–	H18	III	1	South Korea	Korean Peninsula	Chungbuk	–	–	–	–
65	–	KP063146	–	H3	III	1	South Korea	Korean Peninsula	Gangwon	–	–	–	–
66	–	AB085721	–	H1	I	33	Japan	*1	*1	–	–	–	"Allele A"
67	–	AB085722	–	H19	I	11	Japan	*1	*1	–	–	–	"Allele B"
68	–	AB085723	–	H6	II	5	Japan	*1	*1	–	–	–	"Allele C"
69	–	AB085724	–	H20	I	4	Japan	*1	*1	–	–	–	"Allele D"
70	–	AB085725	–	H21	I	2	Japan	*1	*1	–	–	–	"Allele E"
71	–	AB085726	–	H22	I	2	Japan	*1	*1	–	–	–	"Allele F"
72	–	AB085727	–	H23	I	1	Japan	*1	*1	–	–	–	"Allele G"
73	–	AB085728	–	H24	I	1	Japan	*1	*1	–	–	–	"Allele H"
74	–	AB085729	–	H25	I	1	Japan	*1	*1	–	–	–	"Allele I"
75	–	AB085730	–	H26	I	1	Japan	*1	*1	–	–	–	"Allele J"
76	–	AB085731	–	H27	I	1	Japan	*1	*1	–	–	–	"Allele K"
77	–	EF544400	–	H28	CE China	1	CE China	Henan	–	–	–	–	–
78	–	EF544401	–	H29	CE China	1	CE China	Henan	–	–	–	–	–
79	–	EF544406	–	H30	CE China	1	CE China	Henan	–	–	–	–	–
80	–	EF544408	–	H31	CE China	1	CE China	Henan	–	–	–	–	–
81	–	EF544409	–	H32	CE China	1	CE China	Henan	–	–	–	–	–
82	–	EF544417	–	H33	CE China	1	CE China	Henan	–	–	–	–	–
83	–	EU434935	–	H3	III	1	NE China	Jilin	–	–	–	–	–
84	–	EU434936	–	H34	SW China	1	SW China	Yunnan	–	–	–	–	–
85	–	DQ297575	–	H35	SW China	1	SW China	Yunnan	–	–	–	–	–
86	–	DQ351847	–	H3	III	1	NE China	Jilin	–	–	–	–	–
87	–	DQ351848	–	H35	SW China	1	SW China	Yunnan	–	–	–	–	–
88	KUZM17283	LC605946	LC605979	outgroup	outgroup	1	Japan	eastern Japan	Kyoto	35.24	135.49	276	–

Abbreviations: CE, central eastern; NE, northeastern; SW, southwestern.

*1 was listed in Table 2.

### 2.2 DNA extraction, polymerase chain reaction, and sequencing

Genomic DNA was extracted from bat liver tissues preserved in 99% ethanol using the DNeasy Blood & Tissue Kit (Qiagen). MtDNA fragments from the cytochrome *b* and the D‐loop regions were amplified by polymerase chain reaction (PCR) using the newly designed primer pairs RhGluL1 (5'‐AAT CAC CGT TGT ATT TCA AC‐3’) and RhThrH1 (5'‐CTT TTC TGG TTT ACA AGA CC‐3’) for cytochrome *b*, and the universal primer pairs P and E for the D‐loop (Wilkinson & Chapman, 1991). RhGluL1 and RhThrH1 were designed with reference to the complete mitochondrial genome sequence of *R*. *ferrumequinum* (KT779432; Xiao et al., 2017). PCR amplifications were performed in a 12.5 µl reaction volume using an Ex Taq Kit (TaKaRa Bio) and a PCR Thermal Cycler Dice Gradient (TaKaRa Bio), with the following program: an initial denaturing step at 94.0℃ for 5 min; 35 cycles of denaturing at 64.0℃ for 30 s, annealing at 54.5℃ for 30 s, and extension at 72.0℃ for 1 min; and a final extension step at 72.0℃ for 7 min. The PCR products were purified using ExoSAP–IT Express PCR Product Cleanup Reagent (Thermo Fisher Scientific K.K.), and the purified products were sequenced by Macrogen Japan Co.

The sequences were edited and trimmed using GAP 4 (Staden et al., 2003), and aligned using ClustalW (Thompson et al., 1994) in MEGA X version 10.1.8 (Kumar et al., 2018). The sequences were then assembled by eye.

### 2.3 Phylogenetic analysis

A concatenated dataset comprising the cytochrome *b* and the D‐loop fragments was used to estimate the phylogeography of *R*. *nippon*. The D‐loop sequences of individuals with only the cytochrome *b* sequence (*e*.*g*., AB085721–AB085731, Table 1) were treated as missing data. The dataset was partitioned into four parts based on the codon evolutionary rates in cytochrome *b* and the relatively rapid rate in the D‐loop: the first, second, and third triplet positions of cytochrome *b*; and the D‐loop. The haplotype diversity (*h*) and nucleotide diversity (*π*) of the cytochrome *b* and the D‐loop were calculated in DnaSP version 6.12.03 (Rozas et al., 2017).

To assess the divergence times of *R*. *nippon* in Northeast Asia, a time‐calibrated phylogenetic tree of the cytochrome *b* and the D‐loop was estimated by Bayesian inference (BI) in BEAST version 2.6.3 (Bouckaert et al., 2019). BI was based on a partitioned substitution model (K80 for the first, HKY+I for the second, TRN for the third triplet positions of cytochrome *b*, and HKY+G for the D‐loop) selected by the Bayesian information criterion and performed with PartitionFinder version 2.1.1 (Lanfear et al., 2017). BI was run with four Markov Chain Monte Carlo analyses with 100,000,000 iterations and sampling every 50,000 states. Substitution rates of 1.3% per million years for cytochrome *b* (Liu et al., 2016) and 20% for the D‐loop (Kimprasit et al., 2021; Petit et al., 1999), and a generation time of 2 years (Flanders et al., 2009) were used. As a tree prior, the coalescent constant population and strict molecular clock models, and default settings for all other parameters, were selected. The convergence of the runs and the effective sample size was checked in Tracer version 1.7.1 (Rambaut et al., 2018). A consensus tree and posterior probability (PP) were calculated in TreeAnnotator version 2.6.3 (part of the BEAST package) using the maximum clade credibility tree and median heights. The initial 10% of runs was discarded as burn‐in. *R*. *cornutus* (cytochrome *b*, LC605946; D‐loop, LC605979) was used as the outgroup for the BI analyses. A time‐calibrated phylogenetic tree was visualized in FigTree version 1.4.4 (https://github.com/rambaut/figtree/releases), and individuals were gathered into unique haplotypes.

To seek evidence of population growth based on the cytochrome *b* sequences, Tajima's *D* (Tajima, 1989) and Fu's *F*_s_ (Fu, 1997) were tested with 10,000 coalescent simulations in Arlequin version 3.5.2.2 (Schneider et al., 2000). In addition, a median‐joining method (Bandelt et al., 1999) was implemented in NETWORK version 10.2.0.0 (Fluxus Technology, http://www. fluxus‐engineering.com) to construct the maximum parsimony networks of cytochrome *b* and the D‐loop, respectively. The Marine Isotope Stages (MIS) and substages in the following text are based on Oba and Irino (2012).

## 3 RESULTS

### 3.1 Sequencing and haplotypes

First, 1140 bp of cytochrome *b* and 465 bp of the D‐loop were sequenced for all 33 *R*. *nippon* and one *R*. *cornutus* samples. The sequence data were deposited in GenBank (accession nos. LC605914–LC605946 for cytochrome *b*, LC605947–LC605979 for D‐loop). We incorporated the sequence data from GenBank and a previous study, resulting in the analysis of data from a total of 88 sequences (Table 1). In the analyzed cytochrome *b* and D‐loop fragments, 146 and 68 polymorphic sites were detected, and 66 and 33 were parsimony informative, respectively. In Northeast Asia, the nucleotide diversity (π) of the total of 74 sequences based on the cytochrome *b* was 0.00191 (±0.00012), and the diversity of all 27 haplotypes (*h*) was 0.827 (±0.023). The nucleotide diversity of the total of 55 sequences based on the D‐loop was 0.02036 (±0.00308), and the diversity of all 23 haplotypes was 0.890 (±0.034). We joined cytochrome *b* haplotype and D‐loop haplotype as shown in “Haplotype” column in Table 1. For example, “H3‐2” denotes cytochrome *b* haplotype No. 3 and D‐loop haplotype No. 2; “H3” has only cytochrome *b* haplotype No. 3 (lacked the D‐loop). Twenty‐one haplotypes were identified from 32 individuals of *R*. *nippon* newly sequenced in this study. Four haplotypes (H3‐1, H3‐2, H3‐3, and H4‐1) were found in Kumamoto (Kyushu Island): H3 was also found in South Korea and northeastern China (H3, H3‐4, H3‐5, and H3‐6), whereas H4 was unique to Kumamoto. H1 was shared between Kyoto and Ibaraki (H1‐1, H1‐2, H1‐3, H1‐4, J1‐5, and H1‐6), and was consistent with AB085721 (*n* = 33; H3) widely collected from eastern Japan by Sakai et al. (2003). H6 was shared between Fukuoka and Yamaguchi (H6‐1, H6‐2, H6‐3, and H6‐4), and was consistent with AB085723 (*n* = 5). AB085723 were collected from Miyazaki, Aichi, and Shizuoka (Table 2, Figure 1). The other haplotypes were regarded unique to Kyoto (H2‐1), Fukuoka (H5‐1), Hyogo (H7‐1 and H8‐1), and Kagawa (H9‐1, H10‐1, and H11‐1), respectively.

**TABLE 2:** Detail information of sequences examined by Sakai et al. (2003)

No.	Accession	Haplotype	Region	Locality	City	*n*
66	AB085721	H1	eastern Japan	Miyagi	Kashimadai	1
66	AB085721	H1	eastern Japan	Miyagi	Sendai	4
66	AB085721	H1	eastern Japan	Gunma	Matsuida	4
66	AB085721	H1	eastern Japan	Tokyo	Okutama	1
66	AB085721	H1	eastern Japan	Tokyo	Oshima	2
66	AB085721	H1	eastern Japan	Yamanashi	Fuhinomiya	2
66	AB085721	H1	eastern Japan	Shizuoka	Matsuzaki	5
66	AB085721	H1	eastern Japan	Shizuoka	Shizuoka	2
66	AB085721	H1	eastern Japan	Shizuoka	Tenryu	1
66	AB085721	H1	eastern Japan	Niigata	Kashiwazaki	1
66	AB085721	H1	eastern Japan	Toyama	Toyama	3
66	AB085721	H1	eastern Japan	Ishikawa	Oguchi	4
66	AB085721	H1	eastern Japan	Shiga	Taga	1
66	AB085721	H1	eastern Japan	Fukui	Ohno	1
66	AB085721	H1	western Japan	Nagasaki	Tsushima	1
67	AB085722	H19	eastern Japan	Tokyo	Oshima	11
68	AB085723	H6	eastern Japan	Shizuoka	Tenryu	2
68	AB085723	H6	eastern Japan	Aichi	Toyohashi	1
68	AB085723	H6	western Japan	Miyazaki	Miyazaki	2
69	AB085724	H20	eastern Japan	Shiga	Taga	4
70	AB085725	H21	eastern Japan	Miyagi	Kashimadai	2
71	AB085726	H22	eastern Japan	Shizuoka	Tenryu	2
72	AB085727	H23	eastern Japan	Toyama	Toyama	1
73	AB085728	H24	eastern Japan	Aichi	Toyohashi	1
74	AB085729	H25	eastern Japan	Miyagi	Kashimadai	1
75	AB085730	H26	eastern Japan	Fukui	Ohno	1
76	AB085731	H27	eastern Japan	Miyagi	Kashimadai	1

Abbreviations: n, number of individuals.

### 3.2 Time‐calibrated phylogeny in Northeast Asia, determined using cytochrome *b* and the D‐loop

**FIGURE 2:**
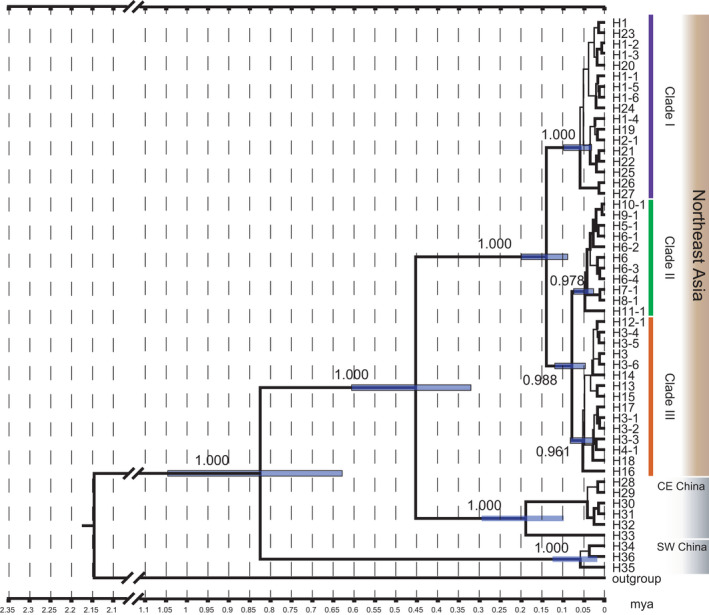
A time‐calibrated phylogenetic tree constructed using Bayesian inference (BI) method based on cytochrome *b* and the D‐loop. A number along each branch is posterior probability based on BI. Blue horizontal bars on nodes indicate 95% HPD intervals for node heights. Branches with posterior probabilities >0.95 are shown as bold lines. Identified haplotypes are listed in Table 1

**FIGURE 3:**
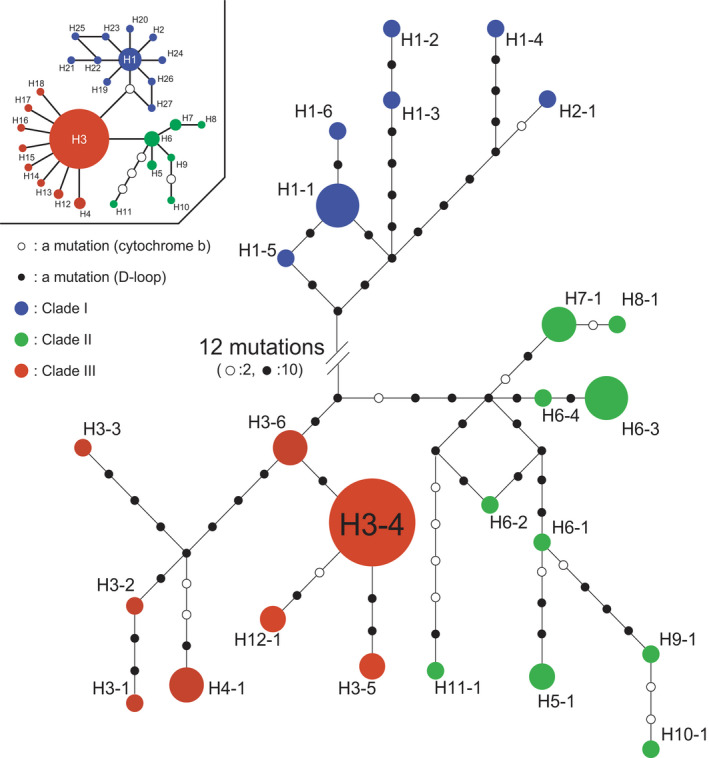
Median‐joining networks based on the mitochondrial cytochrome *b* (upper left) and the D‐loop (middle). Circle size represents haplotype frequency. Identified haplotypes are listed in Table 1

Phylogenetic reconstructions of the BI analyses based on 1605 bp of cytochrome *b* and the D‐loop produced time‐calibrated trees (Figure 2). The monophyly of the Northeast Asian lineage (Figure 2, brown square) was supported by high PP values (1.000). Three clades were supported by high PP values (1.000 for clade I, 0.988 for clades II and III, 0.978 for clade II, 0.961 for clade III). Clade I (Figure 2, blue bar) consisted of individuals from eastern Japan including Kyoto and Ibaraki. Clade II (Figure 2, green bar) consisted of individuals from western Japan including Hyogo, Kagawa, Yamaguchi, and Fukuoka. Clade III (Figure 2, red bar) consisted of individuals from the continent and southern Kyushu including Kumamoto. Two cytochrome *b* and 14 D‐loop mutational steps were found between clade I and clades II and III (Figure 3, between H1‐5 and H3‐6), and at least one cytochrome *b* and six D‐loop mutational steps were found between clade II and clade III (Figure 3, between H3‐6 and H6‐4).

The time‐calibrated phylogeny indicates that *R*. *nippon* in Northeast Asia has experienced at least two divergences after split from Central and East China lineage in 457,700 years ago (320,900–606,400). These divergences are estimated to have occurred during the last two glacial periods [MIS 2.2 (20,000 year ago) and MIS 6.2 (140,000 years ago)] and the interglacial cycles. The divergence between clade I and clades II and III occurred in 142,900 years ago (89,700–200,400 years ago), during the second most recent glaciation in MIS 6.2 and subsequent interglacial periods (MISs 5.2–6.4). The divergence between clade II and clade III occurred in 81,600 years ago (47,300–120,400 years ago), the interglacial period during the last two glacial maxima (MISs 3.0–5.5).

### 3.3 Haplotype network and demographic analysis in Northeast Asia

The obtained median‐joining networks of the cytochrome *b* (Figure 3, upper left) and the cytochrome *b* and the D‐loop (Figure 3) for the samples from Northeast Asia were concordant with the time‐calibrated tree (Figure 2). The cytochrome *b* and the D‐loop‐based network (Figure 3) had three subnetworks corresponding to clade I (blue), clade II (green), and clade III (red).

The haplotype and nucleotide diversities were used to interpret the population's demographic history (Grant & Bowen, 1998). Haplotype and nucleotide diversities based on the D‐loop were high in every clade (*h* > 0.5, *π* > 0.005) because of the high evolutionary rate. By contrast, the haplotype and nucleotide diversities based on cytochrome *b* differed among clades (Table 3). Northeast Asia lineage, clade I, and clade II showed high haplotype diversity and low nucleotide diversity (*h* = 0.827, *π* = 0.00191 for Northeast Asia; *h* = 0.582, *π* = 0.00070 for clade I; *h* = 0.667, *π* = 0.00117 for clade II), suggesting rapid growth and the accumulation of mutations after a bottleneck (Grant & Bowen, 1998). Clade III had low haplotype and nucleotide diversities (*h* = 0.474, *π* = 0.00068), suggesting the recent occurrence of a bottleneck or founder event (Grant & Bowen, 1998). Tajima's *D* test yielded significant negative values for Northeast Asia, clade I, and clade III (*D* = −1.69842, *p* = .017 for Northeast Asia; *D* = −1.53190, *p* = .037 for clade I; *D* = −1.99359, *p* = .007 for clade III), suggesting population expansion. Tajima's *D* value of clade II was not significant (*D* = −1.33113, *p* = .087). Fu's *F*_s_ test also yielded significant negative values for all clades (*F*_s_ = −27.07348, *p* < .001 for Northeast Asia; *F*_s_ = −18.91700, *p* < .001 for clade I; *F*_s_ = −8.40300, *p* < .001 for clade II; *F*_s_ = −17.83900, *p* < .001 for clade III), suggesting population expansion (Table 3).

**TABLE 3:** Genetic diversity and the neutrality tests of *Rhinolophus nippon* in Northeast Asia based on mitochondrial cytochrome *b*

Clade	*n*	*nh*	*π*	*h*	*D*	*p*	*F_S_*	*p*
Northeast Asia	128	27	0.00191	0.827	−1.69842	.017	−27.07348	<.001
Clade I	67	11	0.00070	0.582	−1.53190	.037	−18.91700	<.001
Clade II	21	7	0.00117	0.667	−1.33113	.087	−8.40300	<.001
Clade III	40	9	0.00068	0.474	−1.99359	.007	−17.83900	<.001

Abbreviations: *D*, Tajima's D; *F*_s_, Fu's *F*_s_; *h*, haplotype diversity; *n*, number of individuals; *nh*, number of haplotypes; *p*, p value*; π*, nucleotide diversity.

## 4 DISCUSSION

Our findings support the monophyly of the Northeast Asian lineage of *R*. *nippon*, and it is consistent with previous studies. Moreover, our estimated divergence time for this lineage (320,900–606,400 years ago) was stricter than that of Liu et al. (2016; 220,000–870,000 years ago). This estimate indicates that *R*. *nippon* in Northeast Asia diverged from other East Asian populations in the middle Pleistocene, termed the Chibanian (Cohen et al., 2020) by the International Union of Geological Sciences. The Chibanian (126,000–770,000 years ago) extended from the last geomagnetic reversal to MIS 5 (Dahl‐Jensen et al., 2013).

The Northeast Asian lineage is the most recently diverged lineage of *R*. *nippon* (Flanders et al., 2011; Liu et al., 2016; Rossiter et al., 2007) in the middle Pleistocene (about 430,000 years ago). Rossiter et al. (2007) suggested that the Japanese population experienced genetic isolation and/or founder effects associated with island effects. The results of genetic diversity and the neutrality tests of the Northeast Asian lineage support the occurrence of bottleneck or founder effects associated with the migration of the Northeast Asian ancestors to the Japanese Archipelago, and subsequent population expansion. Within the Northeast Asian lineage, the results of genetic diversity indicates that clade III (the continental and southern Kyushu populations) experienced bottle neck and founder effect more recently than clade I (the eastern Japanese populations) and clade II (the western Japanese populations). The results of neutrality tests indicate all three clades are expanding; however, values of clade II are relatively low. It suggests that the population expansion of clade II is more gentle than other clades. Therefore, in Northeast Asian lineage, it suggests that clade II is the most stable populations, and clade III is the most recently diverged populations.

Flanders et al. (2009) and Liu et al. (2016) suggested that the Japanese populations of *R*. *nippon* were monophyletic clades of the Northeast Asian lineage. Our results, however, elucidate the complex paraphyletic relationships of the Japanese populations. In agreement with Flanders et al. (2009) and Liu et al. (2016), the population from eastern Honshu (the westernmost population is Kyoto) supported monophyly, with clade I as a sister clade to the other Northeast Asian samples. Populations in western Honshu, northern Kyushu, and Shikoku (Hyogo, Yamaguchi, Fukuoka, and Kagawa) form clade II, and those in Kumamoto (southern Kyushu), Jeju, the Korean Peninsula, and northeastern China form clade III. These findings suggest that the Japanese populations are not monophyly.

**FIGURE 4:**
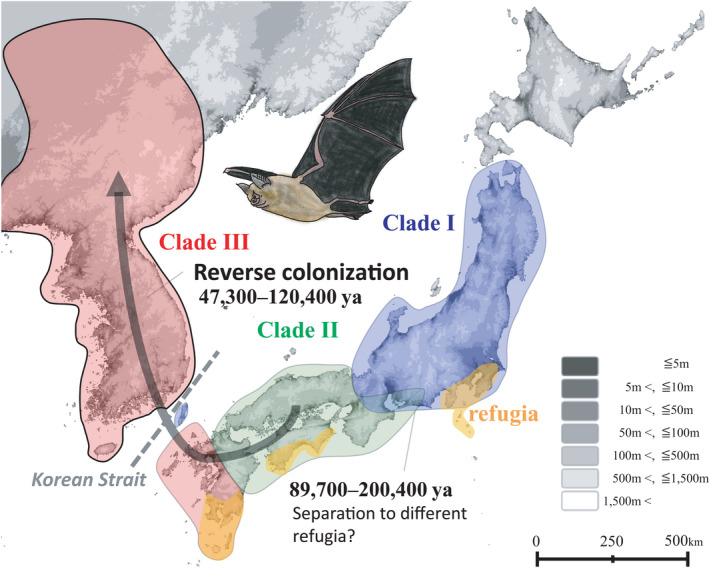
The migration history of *Rhinolophus nippon* in Northeast Asia proposed in this study. Elevation was mapped in grayscale

When continental lineages are imbedded within clades restricted to islands or archipelagos, one can infer “reverse colonization” as the most likely scenario (Bellemain & Ricklefs, 2008). We found that continental clade III is imbedded within Japanese Archipelago clades I and II, suggesting reverse colonization from the Japanese Archipelago to the Eurasian continent. Reverse colonization generates biodiversity and promotes the assembly of continental biota (Patiño et al., 2017). In the Japanese Archipelago, the alpine plant *Primula cuneifolia* recolonized northward to the Kamchatka Peninsula and Alaska after divergence in the less‐glaciated mountains in Japan at the LGM, suggesting that the Japanese Archipelago plays important roles in the diversity and distribution of alpine plants in the northern Pacific region (Ikeda, Yakubov, et al., 2020). Our findings support reverse colonization of bats from the Japanese Archipelago to the continent via the Korean Peninsula (Figure 4). It is certain that the *R*. *nippon* bats flew across between the Japanese Archipelago and the continent. After the last emergence of the land bridge between the Korean Peninsula and the Japanese Archipelago in the middle Pleistocene, the level of the Japan Sea in the glacial periods (MISs 2.2 and 6.2) was 90–100 m lower than in the present, and the Korean Strait width was about one‐third of the present 120 km (Oba & Irino, 2012). The sister species *R*. *ferrumequinum* can fly up to 30 km between summer roosts and hibernacula (Dietz et al., 2009), and *R*. *nippon* in Kyushu recorded the distance 130 km (Sano, 2015). Therefore, *R*. *nippon* seems to be able to fly over the narrow, shallow Korean Strait during a glacial period.

We estimated that clade I in eastern Honshu and clade II in western Honshu bordered in Kinki District (Figure 1). Such a phylogeographic pattern of divergence between eastern and western Honshu is also seen in some Japanese middle and large mammals, such as the sika deer *Cervus nippon* (Ba et al., 2015; Nagata et al., 1999; Yamada et al., 2007), the Asian black bear *Ursus thibetanus* (Ohnishi et al., 2009), and the Japanese macaque *Macaca fuscata* (Kawamoto et al., 2007). Two hypotheses have been proposed to explain the distribution boundary for Japanese middle and large mammals in Kinki district: the multiple‐colonization hypothesis and the refugia hypotheses (Tamate, 2015). *Rhinolophus Nippon* is not distributed along the coast of Far East Russia, the multiple‐colonization hypothesis assuming multiple migrations into the Japanese Archipelago via Korean Peninsula and Sakhalin Island is not applicable. In the refugia hypothesis, organisms evacuated to relatively warm refugia in glacial periods (Bennett & Provan, 2008; Haffer, 1969). In Japan, the southern coasts of Honshu and Shikoku, southern Kyushu, and the southern peripheral islands are considered to have been refugia for plants and animals (Iwasaki et al., 2012; Tamate, 2015; Yamada et al., 2021; Figure 4). We propose that the common ancestor of the Northeast Asian *R*. *nippon* was separated into two or more refugia located in the Japanese Archipelago (Figure 4), suggesting that the refugia hypothesis is applicable to *R*. *nippon*. Moreover, flight dispersal is a crucial difference between bats and other mammals. This difference might enable the reverse colonization from the Japanese Archipelago to the continent. Many terrestrial animals colonized Japan across the land bridge during the glacial periods MIS 16 and 12 (Aimi, 2002; Ba et al., 2015; Dobson & Kawamura, 1998; Millien‐Parra & Jaeger, 1999), and became endemic species. After that, the land bridge had not reappeared, and the Korean Strait prevents the swimming dispersal of terrestrial animals. In contrary, we suggest that bats were able to fly over the strait.

In this study, we targeted the mtDNA cytochrome *b* and D‐loop and revealed that the Japanese populations are paraphyletic and split into three clades. To understand such complicated evolutionary history with revealing demographic and historical background of *R*. *nippon* in Northeast Asia, phylogenetic and population genetic analyses based on other mtDNA and nuclear markers are needed. Additional samples from Tsushima Island are also expected, as Tsushima is located between the Japanese Archipelago and the Korean Peninsula, and close to the Korean Strait. As well Sakai et al. (2003) analyzed mtDNA cytochrome *b* of one individual from Tsushima, which shared a haplotype with the eastern Japan population (AB085721, H1). In Northeast Asia, many phylogeographic studies have been conducted for terrestrial animals, but never for bats which have the unique flight dispersal ability (Sato, 2017). Most organisms migrated in the Japanese Archipelago were adapted to the unique island environment, and became the endemic species. On the other hand, our results indicated that the Japanese populations of *R*. *nippon* would have reverse‐colonized to the continent. There are several other bat species having a distribution area in Northeast Asia similar to *R*. *nippon*. Future studies for demographic and historical background of these bats might bring us the answer why various species can inhabit in Northeast Asia, and propose a new concept that a portion of the biodiversity of Northeast Asian animals was created in the Japanese Archipelago and subsequently returned back to the continent as “reverse colonization.”

## 5 CONCLUSIONS

Northeast Asian lineage of *R*. *nippon* has experienced two divergences at least after the middle Pleistocene. The Japanese populations consist with three main clades and not monophyletic; the continental populations are embedded into one of the clades. Our result suggests that the most likely scenario for Northeast Asian *R*. *nippon* involves reverse colonization from the Japanese Archipelago to the continent. The population transition of *R*. *nippon* within the Japanese Archipelago is consistent with patterns observed for middle and large terrestrial mammals.

## CONFLICT OF INTEREST

The authors declare that they have no conflict of interest.

## AUTHOR CONTRIBUTION

**Yugo Ikeda:** Conceptualization (equal); Data curation (equal); Formal analysis (equal); Investigation (equal); Methodology (equal); Project administration (equal); Resources (equal); Validation (equal); Visualization (lead); Writing – original draft (equal); Writing – review & editing (equal). **Masaharu Motokawa:** Conceptualization (equal); Funding acquisition (equal); Project administration (equal); Visualization (supporting); Writing – review & editing (equal).

## Data Availability

All sequences newly added on this study are usable in GenBank under accession numbers LC605914–LC605979.
